# Mass spectrometric simultaneous quantification of tau species in plasma shows differential associations with amyloid and tau pathologies

**DOI:** 10.1038/s43587-023-00405-1

**Published:** 2023-04-27

**Authors:** Laia Montoliu-Gaya, Andréa L. Benedet, Cécile Tissot, Agathe Vrillon, Nicholas J. Ashton, Wagner S. Brum, Juan Lantero-Rodriguez, Jenna Stevenson, Johanna Nilsson, Mathias Sauer, Nesrine Rahmouni, Gunnar Brinkmalm, Firoza Z. Lussier, Tharick A. Pascoal, Ingmar Skoog, Silke Kern, Henrik Zetterberg, Claire Paquet, Johan Gobom, Pedro Rosa-Neto, Kaj Blennow

**Affiliations:** 1 Department of Psychiatry and Neurochemistry, Institute of Neuroscience & Physiology, Sahlgrenska Academy at the University of Gothenburg, Mölndal, Sweden; 2 Translational Neuroimaging Laboratory, McGill University Research Centre for Studies in Aging, Alzheimer’s Disease Research Unit, Douglas Research Institute, Le Centre intégré universitaire de santé et de services sociaux (CIUSSS) de l’Ouest-de-l’Île-de-Montréal; Department of Neurology and Neurosurgery, Psychiatry and Pharmacology and Therapeutics, McGill University, Montreal, QC Canada; 3 Université de Paris, Cognitive Neurology Center, GHUNord APHP Hospital Lariboisière Fernand Widal, Paris, France; 4 Université de Paris, Inserm UMRS11-44 Therapeutic Optimization in Neuropsychopharmacology, Paris, France; 5 Centre for Age-Related Medicine, Stavanger University Hospital, Stavanger, Norway; 6 Department of Old Age Psychiatry, Maurice Wohl Clinical Neuroscience Institute, King’s College London, London, UK; 7 NIHR Biomedical Research Centre for Mental Health & Biomedical Research Unit for Dementia at South London & Maudsley NHS Foundation, London, UK; 8 Graduate Program in Biological Sciences: Biochemistry, Universidade Federal do Rio Grande do Sul (UFRGS), Porto Alegre, Brazil; 9 Department of Psychiatry, School of Medicine, University of Pittsburgh, Pittsburgh, PA USA; 10 Department of Neurology, School of Medicine, University of Pittsburgh, Pittsburgh, PA USA; 11 Department of Neuropsychiatric Epidemiology, Institute of Neuroscience and Physiology, Sahlgrenska Academy, Centre for Ageing and Health (AgeCap) at the University of Gothenburg, Gothenburg, Sweden; 12 Department of Psychiatry Cognition and Old Age Psychiatry, Sahlgrenska University Hospital, Mölndal, Sweden; 13 Clinical Neurochemistry Laboratory, Sahlgrenska University Hospital, Mölndal, Sweden; 14 Department of Neurodegenerative Disease, Queen Square Institute of Neurology, University College London, London, UK; 15 UK Dementia Research Institute, University College London, London, UK; 16 Hong Kong Center for Neurodegenerative Diseases, Hong Kong, China; 17 UW Department of Medicine, School of Medicine and Public Health, Madison, WI USA

**Keywords:** Alzheimer's disease, Biochemical assays, Ageing

## Abstract

Blood phosphorylated tau (p-tau) biomarkers, at differing sites, demonstrate high accuracy to detect Alzheimerʼs disease (AD). However, knowledge on the optimal marker for disease identification across the AD continuum and the link to pathology is limited. This is partly due to heterogeneity in analytical methods. In this study, we employed an immunoprecipitation mass spectrometry method to simultaneously quantify six phosphorylated (p-tau181, p-tau199, p-tau202, p-tau205, p-tau217 and p-tau231) and two non-phosphorylated plasma tau peptides in a total of 214 participants from the Paris Lariboisière and Translational Biomarkers of Aging and Dementia cohorts. Our results indicate that p-tau217, p-tau231 and p-tau205 are the plasma tau forms that best reflect AD-related brain changes, although with distinct emergences along the disease course and correlations with AD features—amyloid and tau. These findings support the differential association of blood p-tau variants with AD pathology, and our method offers a potential tool for disease staging in clinical trials.

## Abstract (web summary)

A mass spectrometric analysis of plasma tau species identifies phosphorylated tau peptides p-tau217, p-tau231 and p-tau205 with distinct correlations with amyloid and tau pathologies and emergences along the AD continuum.

## Main

The detection of amyloid-β (Aβ) and tau pathologies by cerebrospinal fluid (CSF) and positron emission tomography (PET) biomarkers has enabled an in vivo biological diagnosis of Alzheimer’s disease (AD)^1^. Recently, the availability of ultrasensitive technologies has led to the development of assays capable of measuring the levels of Aβ, tau and neurodegeneration biomarkers in blood^2^. In particular, phosphorylated tau variants (p-tau181 (refs. ^3,4^), p-tau217 (refs. ^5,6^) and p-tau231 (ref. ^7^)) have shown high diagnostic performance in differentiating AD from other neurodegenerative disorders and have been validated against neuropathology^7–9^.

With the emergence of several immunotherapies that efficiently remove Aβ aggregates from the brain, the need for blood biomarkers to facilitate participant recruitment and monitor disease progression in clinical trials is even more pressing. Although a single biomarker (for example, p-tau231 or p-tau217) may work well to identify AD, a biomarker panel may provide further information on disease stage and treatment effects^10^. Moreover, currently available blood p-tau biomarkers appear to have different associations with AD pathology, but a direct comparison of different p-tau variants is hindered by the large heterogeneity in p-tau immunoassays tested in such studies^11–13^. Therefore, a method that systematically measures multiple p-tau and tau species in a single-shot analysis, not dependent on the platform, may give greater insight into stage-specific changes that are critical to monitor pathology changes in drug response and disease progression in clinical management.

**Extended Data Fig. 1:**
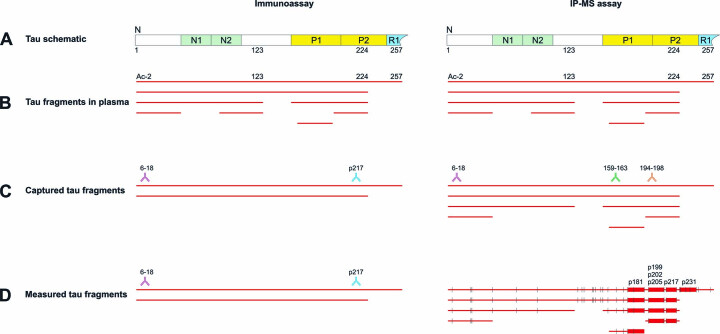
Differences in tau detection in blood by Immunoassays and Immunoprecipitation Mass Spectrometry (IP-MS). (A) Tau is truncated by proteolysis during pathophysiological processing. N-terminal and mid-region tau soluble fragments are generated in the brain and leak into CSF and plasma. Endogenous fragments found in CSF and plasma have been shown to end, among others, at aa123, aa224 and mainly at aa257^14,15^. (B) This creates a pool of distinct fragments to be detected in biofluids. (C) Immunoassays can only capture fragments with the specific length enclosed in the sandwich by the two pair of antibodies. Our IP, by combining 3 antibodies against non-phosphorylated tau -Tau 12 (aa6-18), HT7 (aa159-163) and BT2 (aa194-195) - can capture fragments of different lengths. (D) Further, the captured tau fragments are digested into peptides that are targeted by MS.

Furthermore, studies have shown that tau does not exist as a full-length protein in CSF and blood but, rather, in the form of fragments that contain a mix of N-terminal and mid-region tau^14–17^. Because tau fragments containing specific phosphorylations have different lengths, quatification using immunoassays is limited by the inevitable requirement of the two epitopes being present on the targeted p-tau fragment. This has been shown to influence the quantification of a specific phosphorylation impacting their biological interpretation—for example, in CSF, N-terminal p-tau181 immunoassays report earlier increases in the AD continuum than the mid-region p-tau181 (ref. ^18^). Mass spectrometry (MS) techniques allow simultaneous quantification of several epitopes and enable the detection of a specific p-tau to a broader range of fragments (Extended Data Fig. 1).

The main aim of the current study was to simultaneously quantify the plasma concentrations of six different phosphorylated tau (p-tau181, p-tau199, p-tau202, p-tau205, p-tau217 and p-tau231) and two non-phosphorylated tau peptides using a targeted MS method to investigate their relationship with AD pathology at different stages of disease development. For this, we assessed each biomarkerʼs performance in identifying Aβ pathology using samples from a memory clinic setting (*n* = 157). Then, the biomarkers were evaluated in a well-characterized research cohort with amyloid and tau PET (*n* = 57).

## Results

### Plasma tau species levels across clinical groups

**Fig. 1:**
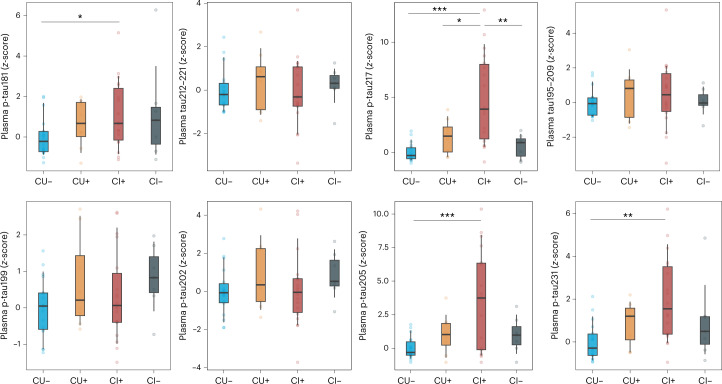
Plasma tau species levels across clinical groups in cohort 2. Box plots of the *z*-scores in plasma p-tau181, tau212–221, p-tau217, tau195–209, p-tau199, p-tau202, p-tau205 and p-tau231 levels quantified by our MS method (*n* = 51). Participants were classified according to cognitive status: CU and CI and amyloid PET uptake positivity (centiloid > 24). To facilitate comparison among peptides, *z*-scores are provided; for absolute concentrations, see Supplementary Fig.3. CU− was used as the reference group. The box plots depict the median (horizontal bar) and 25th to 75th percentiles (hinges), and whiskers indicate 10th and 90th percentiles. Statistical analysis across groups was performed using ANOVA (two-sided), and Tukey contrasts were used to account for multiple comparisons (*P < 0.05, **P < 0.01, ***P < 0.001). Exact significant *P* values: p-tau181 *P*_CU− vs CI+_ = 0.0352; p-tau205 *P*_CU− vs CI+_ < 0.001; p-tau217 *P*_CU− vs CI+_ < 0.001, *P*_CU+ vs CI+_ = 0.0148, *P*_CI+ vs CI−_ = 0.0026; and p-tau231 *P*_CU− vs CI+_ = 0.0015.

**Extended Data Fig. 2:**
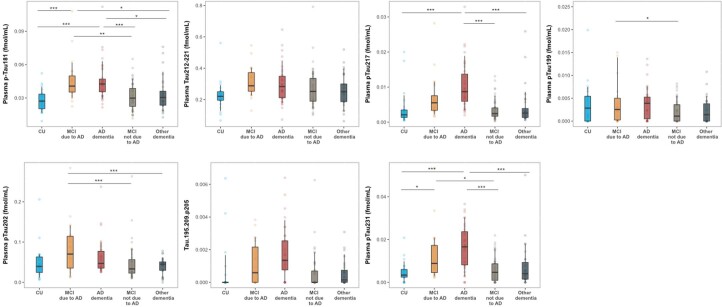
Plasma tau species levels across clinical groups in Cohort 1. Box plots of plasma p-tau181, tau212-221, p-tau217, p-tau199, p-tau202, p-tau205 and p-tau231 levels quantified by our MS method (n=157). Participants were classified according to cognitive status and CSF Aβ42/40 ratio: CU (Cognitively unimpaired), MCI due to AD (Aβ positive Mild Cognitively Impaired), AD dementia, MCI not due to AD (Aβ negative Mild Cognitively Impaired) and other dementia (Aβ negative). The boxplots depict the median (horizontal bar), 25th to 75th percentiles (hinges) and whiskers indicate 10th and 90th percentiles. Statistical analysis across groups was performed using ANOVA (two-sided) and Tukey Contrasts were used to account for multiple comparisons (*P<0.05, **P <0.01, ***P <0.001). Exact significant p-values: P-tau181 *P*_CU vs MCI-AD_<0.001, *P*_CU vs AD_<0.001, *P*_MCI-AD vs MCI-nonAD_=0.0023, *P*_MCI-AD vs Other dementia_=0.025, *P*_AD vs MCI-nonAD_<0.001, *P*_AD vs Other dementia_=0.0113; P-tau217 *P*_CU vs AD_<0.001, *P*_AD vs MCI-nonAD_<0.001, *P*_AD vs Other dementia_<0.001; P-tau199 *P*_MCI-AD vs MCI-nonAD_=0.0425; P-tau202 *P*_MCI-AD vs MCI-nonAD_=0.00164, *P*_MCI-AD vs Other dementia_=0.0063; P-tau231 *P*_CU vs MCI-AD_=0.0156, *P*_CU vs AD_ <0.001, *P*_MCI-AD vs MCI-nonAD_=0.0185, *P*_AD vs MCI-nonAD_<0.001, *P*_AD vs Other dementia_<0.001.

**Extended Data Fig. 3:**
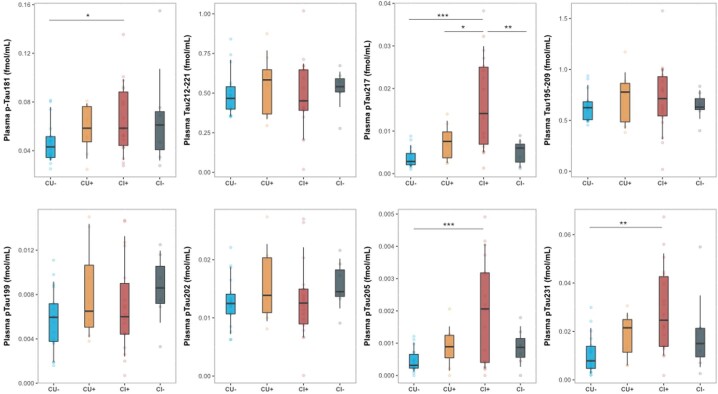
Plasma tau species levels across clinical groups in Cohort 2. Box plots of plasma p-tau181, tau212-221, p-tau217, p-tau199, p-tau202, tau195-209 and p-tau231 levels quantified by our MS method (n=51). Participants were classified according to cognitive status and amyloid PET uptake positivity: CU (Cognitively unimpaired) and CI (Cognitively Impaired). The boxplots depict the median (horizontal bar), 25th to 75th percentiles (hinges) and whiskers indicate 10th and 90th percentiles. Statistical analysis across groups was performed using ANOVA (two-sided) and Tukey Contrasts were used to account for multiple comparisons (*P<0.05, **P <0.01, ***P <0.001). Exact significant p-values: P-tau181 *P*_CU- vs CI+_ = 0.0352; P-tau205 *P*_CU- vs CI+_ <0.001; P-tau217 *P*_CU- vs CI+_ <0.001, *P*_CU+ vs CI+_ = 0.0148, *P*_CI+ vs CI-_ = 0.0026; and P-tau231 *P*_CU- vs CI+_ = 0.0015.

We first used our method to measure samples from a memory clinic population (cohort 1; Supplementary Table 1). Participants were grouped according to clinical diagnosis and CSF biomarker profile in cognitively unimpaired (CU), mild cognitive impairment (MCI), AD dementia, MCI not due to AD and other dementias (Extended Data Fig. 2). We found that p-tau199, p-tau202 and non-phosphorylated tau (tau195–205 and tau212–221) showed no significant differences between AD clinical stages neither in non-AD individuals. Plasma p-tau181, p-tau205, p-tau217 and p-tau231 had significant increases along the AD continuum, being highest in AD dementia except for p-tau181. All p-tau biomarkers were unaltered in MCI and dementia groups without AD pathology. These results were corroborated in cohort 2 (Supplementary Table 2) when participants were classified by cognitive status and amyloid PET positivity (Fig. 1 and Extended Data Fig. 3). Plasma p-tau205, p-tau217 and p-tau231 presented similar mean fold changes (FCs) from CU Aβ-negative (CU−) to CU Aβ-positive (CU+) individuals (FC_p-tau205_ = 1.97, FC_p-tau217_ = 2.05 and FC_p-tau231_ = 2.05). However, in later stages of the disease, p-tau231 levels were not significantly higher in cognitively impaired Aβ-positive (CI+) compared to CU+ (FC_p-tau231_ = 1.5), whereas p-tau205 and p-tau217 presented more dynamic differences (FC_p-tau205_ = 2.26 and FC_p-tau217_ = 2.2). Plasma p-tau181 concentrations were not significantly higher in CU+ compared to CU− (FC_p-tau181_ = 1.24) or CI+ to CU+ (FC_p-tau181_ = 1.14). In all cases, biomarker levels were not significantly different in cognitively impaired Aβ-negative (CI−) or non-AD neurodegenerative diseases (progressive supranuclear palsy (PSP) and frontotemporal dementia (FTD)) compared to CU− (Supplementary Table 3).

### Association of plasma tau species with amyloid PET

**Fig. 2:**
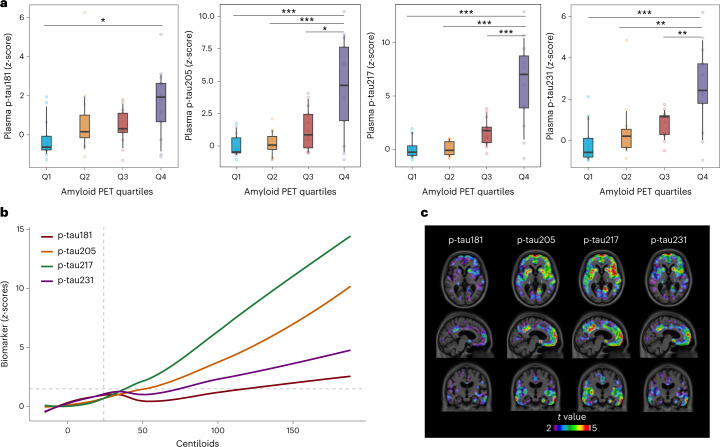
Association of plasma p-tau181, p-tau205, p-tau217 and p-tau231 with amyloid PET. **a**, Box plots of the *z*-scores in the levels of plasma p-tau181, p-tau205, p-tau217 and p-tau231 quantified by MS in participants classified in quartiles according to their amyloid PET uptake (Q1 = (−Inf, 1.29); Q2 = (1.29, 1.7); Q3 = (1.7, 2.45); Q4 = (2.45, Inf)) (*n* = 51). The reference group was Q1. To facilitate comparison among peptides, *z*-scores are provided; for absolute concentrations, see Supplementary Fig. 4. The box plots depict the median (horizontal bar) and 25th to 75th percentiles (hinges), and whiskers indicate 10th and 90th percentiles. Statistical analysis across groups was performed using age-adjusted and sex-adjusted ANOVA (two-sided), and Tukey contrasts were used to account for multiple comparisons (*P < 0.05, **P < 0.01, ***P < 0.001). Exact significant *P* values: p-tau181 *P*_Q1 vs Q4_ = 0.0118; p-tau205 *P*_Q1 vs Q4_ < 0.001, *P*_Q2 vs Q4_ < 0.001, *P*_Q3 vs Q4_ = 0.0015; p-tau217 *P*_Q1 vs Q4_ < 0.001, *P*_Q2 vs Q4_ < 0.001, *P*_Q3 vs Q4_ < 0.001; p-tau231 *P*_Q1 vs Q4_ < 0.001, *P*_Q2 vs Q4_ = 0.0029, *P*_Q3 vs Q4_ = 0.0095. **b**, LOESS plots showing the smoothed relationship between biomarker levels and accumulation of amyloid pathology. Biomarker levels are presented as *z*-scores, using the average biomarker concentrations of the CU− group as reference. Amyloid pathology is indexed here by global amyloid PET SUVR, which was converted to the centiloid scale for comparison purposes. Horizontal dashed line indicates two *z*-scores, a likely indication of biomarker abnormality. Vertical dashed line indicates centiloid 24, the cutoff of amyloid positivity reported for TRIAD cohort. **c**, Voxel-wise associations of p-tau181, p-tau205, p-tau217 and p-tau231 with amyloid PET uptake. Unadjusted parametrical *t*-statistical maps depict the results of the association between plasma biomarkers and amyloid PET at the voxel level. Voxels with *t* > 3 have a significant association between the variables evaluated (maps adjusted for multiple comparisons are presented in Extended Data Fig. 5).

**Extended Data Fig. 4:**
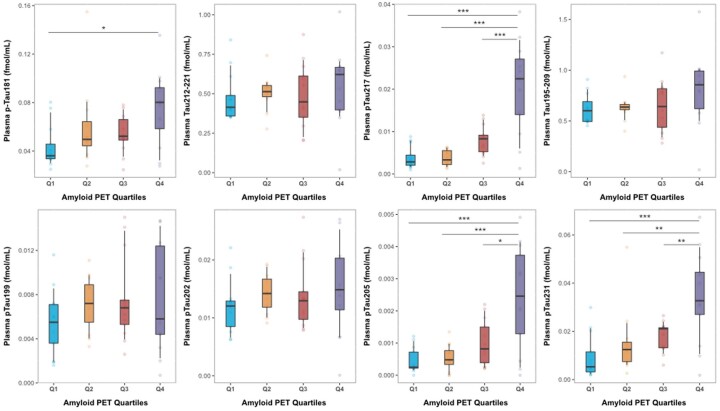
Plasma tau species across Amyloid PET quartiles in Cohort 2. Box plots of plasma p-tau181, tau212-221, p-tau217, p-tau199, p-tau202, tau195-209 and p-tau231 levels in relation to amyloid PET (n=51). Participants were grouped in quartiles according to their levels of amyloid PET uptake (Q1=(-Inf,1.29]; Q2=(1.29,1.7]; Q3=(1.7,2.45]; Q4=(2.45, Inf]). The boxplots depict the median (horizontal bar), 25th to 75th percentiles (hinges) and whiskers indicate 10th and 90th percentiles. Statistical analysis across groups was performed using ANOVA (two-sided) and Tukey Contrasts were used to account for multiple comparisons (*P<0.05, **P<0.01, ***P<0.001). Exact significant p-values: P-tau181 *P*_Q1 vs Q4_ =0.0118; P-tau205 *P*_Q1 vs Q4_ <0.001, *P*_Q2 vs Q4_ <0.001, *P*_Q3 vs Q4_ = 0.0015; P-tau217 *P*_Q1 vs Q4_ <0.001, *P*_Q2 vs Q4_ <0.001, *P*_Q3 vs Q4_ <0.001; P-tau231 *P*_Q1 vs Q4_ <0.001, *P*_Q2 vs Q4_ = 0.0029, *P*_Q3 vs Q4_ = 0.0095.

Plasma p-tau205, p-tau217 and p-tau231 were the biomarkers with higher correlations with Aβ PET global standardized uptake value ratio (SUVR) (p-tau205, *r* = 0.52, *P* < 0.001; p-tau217, *r* = 0.70, *P* < 0.001; and p-tau231, *r* = 0.60, *P* < 0.001), followed by p-tau181 (*r* = 0.42, *P* = 0.002) (Supplementary Table 4). When stratifying amyloid PET into quartile (Q) groups, although all three phosphos were statistically similar, the same trend as before was observed with disease stage. Plasma p-tau205, p-tau217 and p-tau231 presented higher levels in Q3 of amyloid PET uptake compared to Q1 (FC_p-tau205_ = 2.24, FC_p-tau217_ = 2.1 and FC_p-tau231_ = 1.90), but p-tau205 and p-tau217 showed higher concentrations in Q4 (FC_p-tau205_ = 5.43 and FC_p-tau217_ = 5.36) than p-tau231 (FC_p-tau231_ = 3.65) (Fig. 2a and Extended Data Fig. 4). Changes in the levels of p-tau species in relation to brain amyloid accumulation also suggested that p-tau181 and p-tau231 increase earlier than p-tau205 and p-tau217, although they all changed close to amyloid load PET uptake abnormality (centiloid < 24) (Fig. 2b). Plasma p-tau217 and p-tau205 showed higher mean FCs with amyloid deposition than p-tau231 and p-tau181. Voxel-based analysis indicated an overall topographic overlap of the brain regions in which plasma biomarkers are associated with [^18^F]AZD4694 retention, mostly across the posterior cingulate, precuneus, prefrontal and temporal cortices. However, in this dataset, p-tau217 and p-tau205 showed wider voxel associations with larger *t* values, particularly when compared to p-tau181 (Fig. 2c).

### Association of plasma tau species with tau PET

**Fig. 3:**
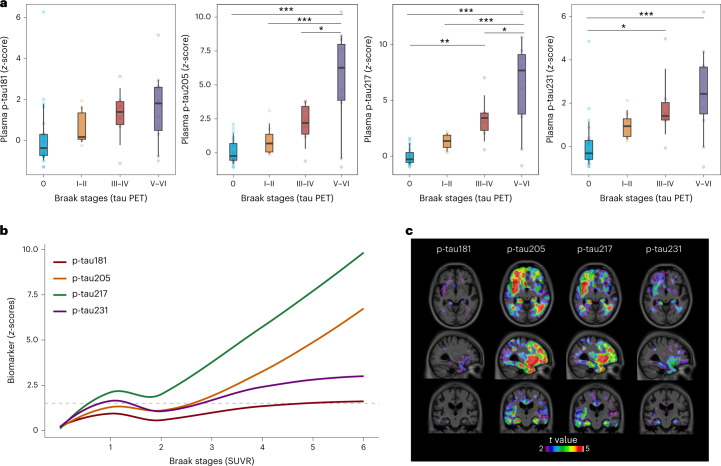
Association of plasma p-tau181, p-tau205, p-tau217 and p-tau231 with tau PET. **a**, Box plots of the *z*-scores in the levels of plasma p-tau181, p-tau205, p-tau217 and p-tau231 quantified by MS in participants according to regional spreading of tau classified by Braak stages (*n* = 51). The reference group was Braak 0. To facilitate comparison among peptides, *z*-scores are provided; for absolute concentrations, see Supplementary Fig. 6. The box plots depict the median (horizontal bar) and 25th to 75th percentiles (hinges), and whiskers indicate 10th and 90th percentiles. Statistical analysis across groups was performed using age-adjusted and sex-adjusted ANOVA (two-sided), and Tukey contrasts were used to account for multiple comparisons (*P < 0.05, **P < 0.01, ***P < 0.001). Exact significant *P* values: p-tau205 *P*_0 vs V–VI_ < 0.001, *P*_I–II vs V–VI_ < 0.001, *P*_III–IV vs V–VI_ = 0.0119; p-tau217 *P*_0 vs V–VI_ < 0.001, *P*_0 vs III–IV_ = 0.0082, *P*_I–II vs V–VI_ < 0.001, *P*_III–IV vs V–VI_ = 0.0468; p-tau231 *P*_0 vs V–VI_ < 0.001, *P*_0 vs III–IV_ = 0.050. **b**, LOESS plots show the smoothed relationship between biomarker levels and Braak stages. Biomarker levels are presented as *z*-scores, using the average biomarker concentrations of the CU− group as reference. Horizontal dashed line indicates two *z*-scores, a likely indication of biomarker abnormality. **c**, Voxel-wise associations of p-tau181, p-tau205, p-tau217 and p-tau231 with tau PET uptake. Unadjusted parametrical *t*-statistical maps depict the results of the association between plasma biomarkers and tau PET at the voxel level. Voxels with *t* > 3 have a significant association between the variables evaluated (maps adjusted for multiple comparisons are presented in Extended Data Fig. 7).

**Extended Data Fig. 6:**
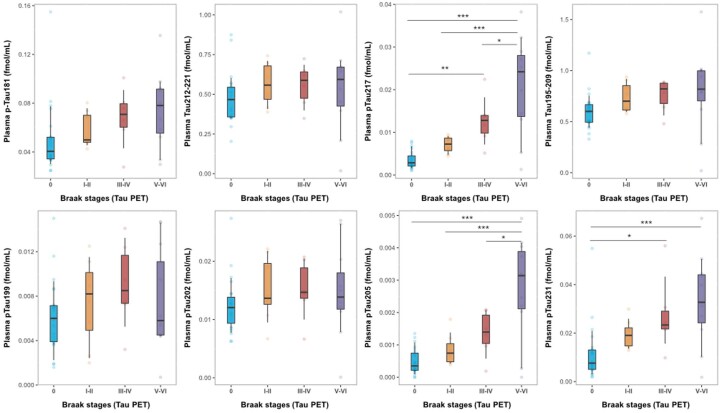
Plasma tau species across Braak stages in Cohort 2. Box plots of plasma p-tau181, tau212-221, p-tau217, p-tau199, p-tau202, tau195-209 and p-tau231 levels in relation to amyloid PET (n=51). Participants were grouped in quartiles according to regional spreading of tau classified by Braak stages. The boxplots depict the median (horizontal bar), 25th to 75th percentiles (hinges) and whiskers indicate 10th and 90th percentiles. Statistical analysis across groups was performed using ANOVA (two-sided) and Tukey Contrasts were used to account for multiple comparisons (*P<0.05, **P<0.01, ***P<0.001). Exact significant p-values: P-tau205 *P*_0vsV-VI_ <0.001, *P*_I-II vs V-VI_ <0.001, *P*_III-IV vs V-VI_ = 0.0119; P-tau217 *P*_0 vs V-VI_ <0.001, *P*_0 vs III-IV_ = 0.0082, *P*_I-II vs V-VI_ <0.001, *P*_III-IV vs V-VI_ = 0.0468; P-tau231 *P*_0 vs V-VI_ <0.001, *P*_0 vs III-IV_= 0.050.

Correlations of plasma tau peptides with tau PET uptake indicated that p-tau205, p-tau217 and p-tau231 were the site-specific phosphorylations with higher associations (p-tau205, *r* = 0.49, *P* < 0.001; p-tau217, *r* = 0.58, *P* < 0.001; p-tau231, *r* = 0.43, *P* < 0.001) but not significant for p-tau181, p-tau199 and p-tau202 (Supplementary Table 4). Plasma p-tau217 and p-tau231 showed the first significant differences in Braak III–IV (*P* = 0.047 and *P* = 0.050, respectively), whereas, for p-tau205, the difference was significant only at Braak V–VI (*P* < 0.001). Interestingly, p-tau205 and p-tau217 levels were significanly higher in Braak V–VI compared to Braak III–IV (*P* = 0.012 and *P* = 0.008, respectively) but not for p-tau231 (*P* = 0.79) or other p-tau species (Fig. 3a and Extended Data Fig. 6). Visual inspection of locally estimated scatterplot smoothing (LOESS) analyses highlighted plasma p-tau217 as the biomarker with the steepest slope in relation to tau pathology stage and p-tau205 as the latest one to become abnormal, between Braak II and Braak III (Fig. 3b). Voxel-wise analyses showed associations between plasma biomarkers and [^18^F]MK6240 uptake in the superior, middle and inferior temporal gyri as well as in the entorhinal cortex and hippocampus bilaterally, which were particularly evident for p-tau205 and p-tau217 (Fig. 3c).

### Relation of plasma tau species with amyloid and tau pathologies

**Fig. 4:**
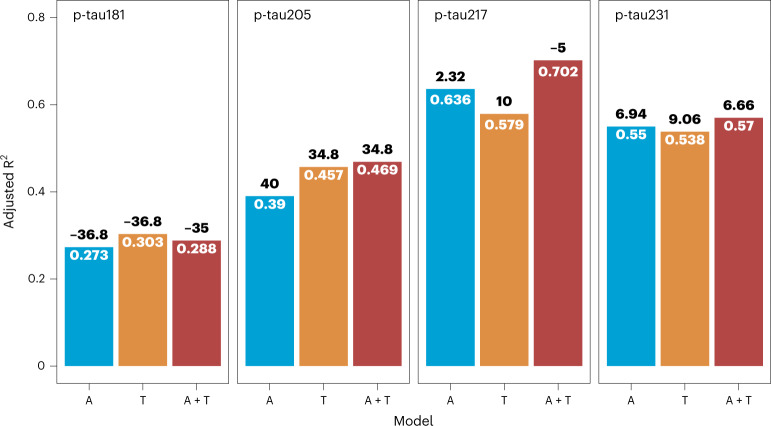
Amyloid and tau pathologies regression models for plasma p-tau181, p-tau205, p-tau217 and p-tau231 quantified by targeted MS. R^2^ and AIC are indicated for the different regression models, using only amyloid PET signal (A: indexed by global amyloid PET SUVR), only tau PET signal (T: indexed by global tau PET SUVR) or both amyloid and tau (A+T). The panel shows adjusted R^2^ for age and sex (inside the bars), together with AIC (above the bars) for each model (*n* = 51).

To characterize the contribution of plasma tau species to both amyloid and tau signals, we performed regression models using amyloid PET (A: indexed by global amyloid PET SUVR), tau PET (T: indexed by global tau PET SUVR) or both amyloid and tau (A+T) (Fig. 4). Plasma p-tau205, p-tau217 and p-tau231 were the phosphorylation sites that better associated with the imaging findings. For plasma p-tau231, the lowest Akaike information criterion (AIC) values were observed in the A and A+T models (AIC_A_ = 6.94 and AIC_A+T_ = 6.66), with similar regression coefficients (R^2^_A_ = 0.55 and R^2^_A+T_ = 0.57). This suggests that amyloid PET SUVR (A) is the model that better explains plasma p-tau231 for being the simplest. Plasma p-tau217 was mainly mediated by A+T (R^2^ = 0.702, AIC = −5). Plasma p-tau205 presented the same AIC for T and A+T (AIC_T_ = 34.8 and AIC_A+T_ = 34.8), with similar regression values (R^2^_T_ = 0.457 versus R^2^_A+T_ = 0.469), indicating that T is the main mediator of p-tau205. Finally, we determined the ability of the tau species to identify amyloid and tau pathologies (Supplementary Table 5). Plasma p-tau217 presented the highest accuracy distinguishing amyloid PET-positive from PET-negative individuals (area under the curve (AUC) = 0.85 (95% confidence interval (CI): 0.74–0.95)), further increased when using the phospho/non-phospho ratio p-tau217/212–221 (AUC = 0.94 (95% CI: 0.87–1)). Plasma p-tau217 and p-tau205 showed similar performances to discriminate Braak I–IV versus Braak V–VI (AUC = 0.81 (95% CI: 0.59–1) and AUC = 0.81 (95% CI: 0.58–1), respectively), improved when using the ratio p-tau217/tau212–221 (AUC = 0.96 (95% CI: 0.63–1), DeLongʼs test *P* = 0.04) but not significantly superior for the ratio p-tau205/tau195–209 (AUC = 0.84 (95% CI: 0.63–1), DeLongʼs test *P* = 0.12).

## Discussion

Plasma p-tau biomarkers have shown high value in detecting AD in patients with cognitive complaints and predicting the future development of AD^12^. Thus, because of their additional value of being less invasive and cost-effective, they are now being used as recruitment and treatment monitoring tools in clinical trials for anti-Aβ therapies^19^ and will soon be implemented for diagnostic purposes in clinical practice^19^. In this study, by using an MS method to simultaneously quantify six different phosphorylated and two non-phosphorylated tau peptides in plasma, we show that not all tau phosphorylations detected in blood reflect the same brain pathological changes and evolve in the same direction with proxies of disease progression. Our results support a previous study in autosomal dominant AD using an MS method, which found that hyperphosphorylation at specific sites of the tau protein is a dynamic process with predictable progression in the phosphorylation pattern when measured in CSF^20^. Here we demonstrate that this dynamic process can also be detected in plasma across the full clinical spectrum of sporadic AD.

Our results corroborate previous observations that p-tau231 presents an early change in preclinical AD, emerging before amyloid PET abnormality^7,21^. Furthermore, we observed that p-tau231 levels are not further increased in individuals with more advanced symptomology, which verifies immunoassay findings^10^. This could be explained by the strong association of p-tau231 with amyloid pathology, increasing with the early accumulation of Aβ but plateauing in later stages when amyloid deposition stabilizes^22^. Plasma p-tau181 showed similar trends and emergence as p-tau231, but the mean FCs of this biomarker were more subtle, showing weaker and non-significant associations with amyloid and tau pathologies, respectively. Several studies comparing different plasma p-tau assays have reported p-tau181 having lower accuracies in detecting AD compared to p-tau217 and p-tau231 (refs. ^12,13,23,24^). Another possible explanation might be a limitation of the MS technique. In particular, the phosphorylation at position Thr181 prevents digestion by trypsin between residues Lys180 and Thr181, which allows the detection of the phosphorylated peptide 175–190 but not the non-phosphorylated version of the exact same peptide. This was also observed previously by us and others in CSF^17,25^. It is possible that trypsin cleaves a small percentage of the phosphorylated peptide, which would be undetected and lead to an underestimation of this phosphorylation. In addition, we previously showed that an endogenous tau peptide (tau 175–190) is present in CSF, in both phosphorylated and non-phosphorylated forms, but does not change in AD^26^. It is likely that this endogenous peptide is also secreted to plasma and, because it is the same as the one generated by trypsin, is, thus, possible to affect results.

In contrast, p-tau217 has been shown as the p-tau that exhibits larger FCs in symptomatic AD phases in CSF^27^ and plasma^5,9,28^. We corroborate that plasma p-tau217 presents continuous and higher increases along the AD continuum^10^ and better associates with amyloid and tau pathologies determined by PET^5,6,28^. By simultaneously measuring different p-tau species, we observed that p-tau217 performs superiorly to other p-tau species identifying both pathologies. Furthermore, our results support previous findings suggesting that plasma p-tau217 is a dynamic biomarker associated with amyloid pathology in early stages and with tau burden later, specifically reflecting the progression of ADʼs main biological features^6^.

To our knowledge, this is the first study reporting the quantification of p-tau205 in blood. Although its accuracy detecting amyloid pathology was inferior to p-tau217 and p-tau231, this epitope (1) performed similarly to p-tau217 in identifying advanced tau pathology, (2) was the only p-tau biomarker that was better explained by the tau PET signal and (3) presented the latest changes to abnormality in relation to Braak staging. Postmortem staging of neurofibrillary tangles (NFTs) has traditionally been performed by immunostaining using the AT8 antibody, which reacts against hyperphosphorylated tau at positions 202/205 (ref. ^29^), making the in vivo quantification of this biomarker an important tool for examining neuropathological associations. In addition, findings in autosomal dominant AD suggest that CSF p-tau205 levels increase with the beginning of neuronal dysfunction, years later than the rise in p-tau217 (ref. ^20^). Altogether, these results point to p-tau205 as a late biomarker possibly linked to tau burden. Considering the recent success of the TRAILBLAZER-2 donanemab trial^30^ based on recruiting participants with intermediate tau PET burden, we postulate that the window between p-tau217 and p-tau205 could be used to better identify this group.

Finally, plasma p-tau199, p-tau202 and the non-phosphorylated tau species tau195–209 and tau212–221 did not change significantly along the AD continuum. No previous studies have assessed the levels of p-tau199 in blood, and plasma p-tau202 concentrations have shown no correlations with CSF measures^15^. Assays targeting non-phosphorylated versions of tau—or total-tau (t-tau)—although very successful in CSF, have shown limited accuracy in blood^15^. This is probably due to the contribution of peripheral tau^31^ to the quantification masking the measurement of brain-specific tau, which has been estimated to account for approximately 20% of the total tau protein detected in blood^15^. Despite this, the ratio phosphorylated/non-phosphorylated peptides for p-tau217, as shown here and previously^13,15^, performs better identifying AD pathology than the peptide alone. However, in this work, the ratio was not significantly better for p-tau205. Future studies should address the biological significance of these ratios considering the fragmentation pattern of tau in blood and the contribution of central nervous and peripheral nervous system tau to these quantifications, as well as to which degree the benefit of normalization by using such ratios is due to minimizing methodological variability.

A few methodological factors should be considered on the interpretations of our results. Although this study introduces a method for the simultaneous quantification of different plasma tau species, the number of samples with neuroimaging data available was limited. Furthermore, assessment of tau pathology in the brain using PET quantifies aggregated tau in NFTs, whereas the tau pool detected in plasma is composed of soluble forms of the protein, which could be a limitation when establishing correlations. Differences in the information provided by fluid biomarkers and imaging tracers could have also affected the relationship of the plasma tau species with amyloid pathology. Amyloid PET uptake becomes abnormal later than fluid Aβ^32,33^, and close to fluid p-tau^7,34^, explaining why plasma tau biomarkers showed similar trends in early amyloid PET uptake. In addition, the analysis of the accuracy of the ratio phosphorylated/non-phosphorylated peptides was limited to p-tau217 and p-tau205, because our panel lacked the non-phosphorylated versions of p-tau181 and p-tau231 due to technical caveats discussed before. Finally, larger studies are needed to confirm the differential associations of these biomarkers with amyloid and tau pathologies, and longitudinal studies should address their prognostic value over clinical progression as well as their evolution during the disease course.

In conclusion, we have developed a MS method to simultaneously quantify six different phosphorylated (p-tau181, p-tau199, p-tau202, p-tau205, p-tau217 and p-tau231) and two non-phosphorylated (tau195–209 and tau212–221) tau peptides in plasma. Our results indicate that p-tau217, p-tau231 and p-tau205 are the plasma tau forms that best reflect AD-related brain pathological changes although with different emergence along the AD continuum and associations with amyloid and tau pathologies. Plasma p-tau231 was found to be the earliest p-tau biomarker; p-tau217 showed the highest FCs and diagnostic performance; and p-tau205 was observed to become abnormal in later stages of the disease. A comprehensive understanding of the pathological information that each blood p-tau reflects is paramount to decide which biomarker to use in each stage of the disease and to guarantee a correct read-out in clinical trials for anti-Aβ and anti-tau therapies.

## Methods

### Study participants

#### Discovery cohort

We evaluated the performance of the plasma tau MS method by analyzing plasma from 24 participants in the Gothenburg H70 Birth Cohort Studies (12 AD and 12 controls). Participants were selected and grouped according to CSF biomarker values (Aβ42/40 ratio < 0.62 and ptau > 60 pg ml^−1^).

#### Cohort 1

A total of 157 patients from the Cognitive Neurology Center, Lariboisière Fernand Widal Hospital, Université Paris Cité, was included in the study. Patients went through a comprehensive neurological examination, neuropsychological assessment and CSF and plasma biomarker analysis. Cognitive follow-up data were acquired for an average time of 6 months. Samples from patients were grouped according to the clinical diagnosis at the memory clinic and biological CSF marker profile: controls (*n* = 23), AD–MCI (*n* = 24), AD (*n* = 27), non-AD MCI (*n* = 50) and other dementias (*n* = 33).

#### Cohort 2

Participants from the Translational Biomarkers of Aging and Dementia (TRIAD) cohort who had amyloid and tau PET imaging as well as plasma volume available at the time of the MS experiments were included in the study (*n* = 57). The TRIAD cohort is well characterized in terms of biomarker and clinical data and contains participants ranging from CU young (<30 years of age) and older adult (>50 years of age) individuals to patients with MCI and AD dementia. CU participants had a Mini-Mental State Examination (MMSE) score >24 and a Clinical Dementia Rating (CDR) score of 0. MCI participants had a CDR score of 0.5—subjective and objective impairments in cognition but preserved activities of daily living. Patients with AD dementia had a CDR score ≥0.5 and met the National Institute on Aging and Alzheimerʼs Association criteria for AD determined by diagnostic^35,36^. For the purposes of this study, CU, MCI and AD dementia participants were grouped as CU (young and older adults) and CI (MCI and dementia) as well as according to their Aβ status (based on Aβ PET visual rating) as Aβ-positive (+) or Aβ-negative (−). The resulting clinical and biomarker-defined groups were: 18 CU−, 8 CU+, 18 CI+ and 7 CI−.

All participants provided written informed consent, and all three studies were approved by their regional ethics committee. REB approval for TRIAD IUSMD-16-60 2021 (Centre intégré universitaire de santé et de services sociaux (CIUSSS) de l'Ouest-de-l'Île-de-Montréal–Mental Health and Neuroscience). The Paris Lariboisière cohort was approved by the ethic committee of Bichat University, Paris, France (CEERB GHU Nord n°10-037).

### Plasma tau MS analysis

MS detection of phosphorylated and non-phosphorylated tau peptides was performed by adapting a previous method for CSF to blood^25^. In this case, EDTA plasma samples (1 ml) were thawed, vortexed for 30 s at 2,000 r.p.m. and spun down for 10 min at 4,000*g*. Tau protein was extracted by IP using beads (Dynabeads M-280 sheep anti-mouse IgG, Thermo Fisher Scientific, 11202D) cross-linked with a combination of antibodies targeting non-phosphorylated tau: Tau12 (BioLegend, 806501), HT7 (Thermo Fisher Scientific, MN1000) and BT2 (Thermo Fisher Scientific, MN1010). Antibodies were conjugated to the beads at a concentration of 4 µg antibody/50 µl beads. Automated IP was performed using the KingFisher Flex System (Thermo Fisher Scientific). Samples were incubated with the beads for 2 h at room temperature, followed by washes with PBS, PBS 0.05% Triton X-100, PBS and 50 mM ammonium bicarbonate (AMBIC) and elution with 0.5% formic acid. Quality control samples (which were a pool of several plasma samples) and recombinant tau (0.001 µg per sample) were included in each plate as IP control and to monitor intensity signal for normalization purposes. Further tau enrichment was performed by adding perchloric acid (15 µl, 60% v/v) to the samples, which induces precipitation of the vast majority of proteins but not tau. After centrifugation at 3,000*g* for 30 min at 4 °C, supernatants were transferred to a 96-well SPE plate (Oasis PRiME HLB 96-well µElution plate, 3 mg of sorbent per well; Waters) and desalted. The SPE plate was washed with 2 × 200 µl of 5% methanol (v/v) and eluted into a microtiter plate with 200 µl of 50% acetonitrile and 0.1% trifluoroacetic acid. Then, samples were speed-vac-dried. Tryptic digestion was performed by resuspending the samples with trypsin solution (sequencing grade, Promega) (0.1 µg per sample at a concentration 2.5 µg/ml^−1^ in 50 mM AMBIC) and incubating overnight at 37 °C. After 18 h, proteolysis was quenched with trifluoroacetic acid (TFA) final concentration 0.1%, and samples were lyophilized and stored at −20 °C.

For liquid chromatography–mass spectrometry (LC–MS) analysis, samples were resuspended in 50 µl of 0.01% TFA and run in singlicates. LC settings were the same as previously described^14^. MS analysis was performed on a hybrid Orbitrap mass spectrometer (Fusion Tribrid, Thermo Fisher Scientific, for the discovery and memory clinic cohorts and Lumos for TRIAD), fitted with an EasySpray nano-ESI ion source. The mass spectrometer was operated in the positive ion mode, with the following settings for the parallel reaction monitoring (PRM) scan: Activation Type: HCD; Detector Type: Orbitrap; Orbitrap Resolution: 60,000; Scan Range: 250–1,200; RF Lens: 30%; Easy-IC: On; Isolation Type: Quadrupole; and Isolation Window: 0.7 *m*/*z*. Maximum Injection Time, Normalized AGC Target, Optimal Collision Energy and FAIMS Voltage were determined experimentally for each peptide. The endogenous tryptic peptides targeted in this study are shown in Supplementary Table 6. Heavy-labeled AQUA peptide standards were prepared in a mix with adjusted concentrations for each peptide and spiked in during the sample preparation (Supplementary Table 7). LC–MS data were acquired using Xcalibur 4.5 and Tune 3.5 software (Thermo Fisher Scientific) and analyzed with Skyline 22.2 software (McCoss Laboratory, University of Washington). The analysis of the plasma samples was performed blinded to any participant information.

**Extended Data Fig. 8:**
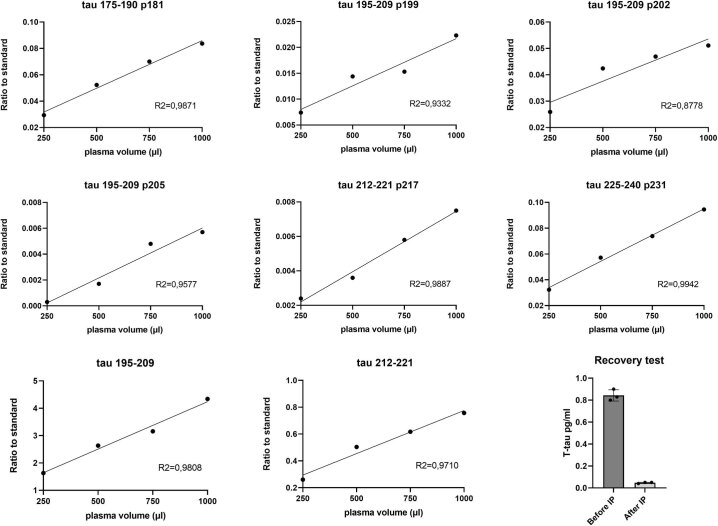
Linearity and recovery tests for the plasma tau IP-MS method. Linearity: plasma samples of different volumes (250, 500, 750 and 1000µl) were prepared and analyzed with the method. Simple linear regressions were performed (R^2^) and are shown for each peptide. Recovery: total-tau protein concentration before and after immunoprecipitation (IP) performed for sample preparation before MS analysis (n=3). Data shown as mean ± SD.

**Extended Data Fig. 9:**
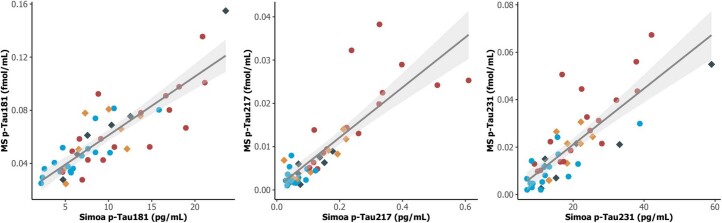
Correlation between IP-MS and Simoa quantification for p-tau181, p-tau217 and p-tau231 in the TRIAD cohort. Participants are color coded according to cognitive status: CU (Cognitively unimpaired) and CI (Cognitively Impaired), and amyloid PET uptake positivity (Centiloid>24): blue (CU-), orange (CU+), red (CI+) and dark grey (CI-). Spearman rho correlation were: Rp-tau181=0.83 (P<2.2x10-16), Rp-tau217=0.82 (P<2.2x10-16) and Rp-tau231=0.83 (P<2.2x10-16). P-values two-sided.

**Extended Data Fig. 10:**
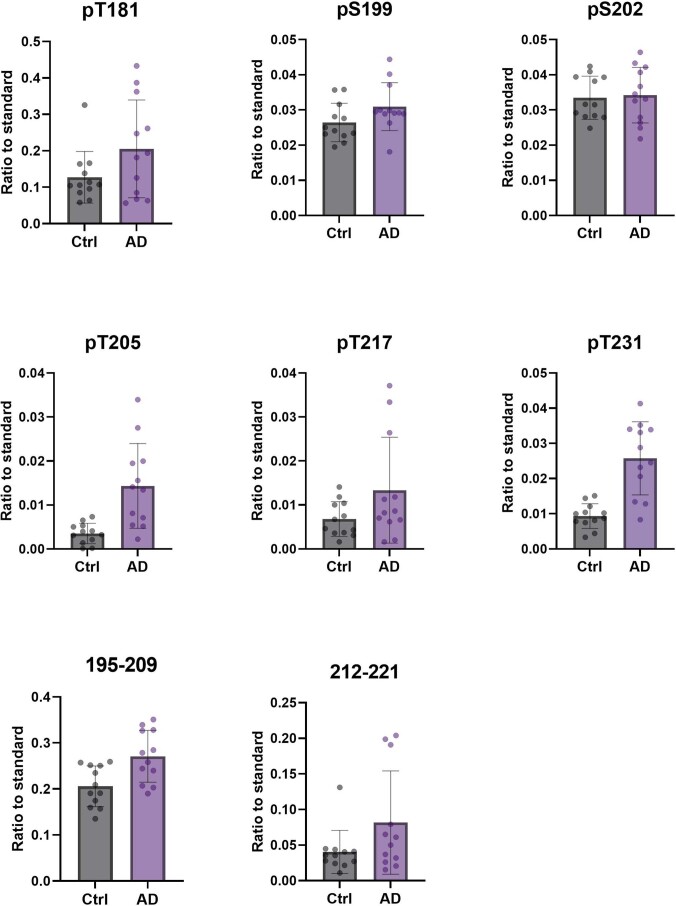
Plasma tau peptides quantification in the Discovery Cohort. Plasma samples from 24 participants in the H70 Clinical Studies (12 AD and 12 controls (Ctrl)) were analyzed with the IP-LC/MS method. Samples were selected according to their corresponding CSF biomarker values: Aβ42/40 ratio < 0.062 and p-tau > 60 ng/L. Data shown as mean ± SD.

The reproducibility of the method was tested by measuring the same sample repeatedly and at spaced times (Supplementary Table 8). Linearity was assessed by preparing plasma samples of different volumes (250 µl, 500 µl, 750 µl and 1,000 µl) and analyzed with the method (Extended Data Fig. 8). A recovery test was performed to guarantee that beads were not saturated during the IP and there was no tau protein remaining in the supernatant after the sample preparation. The total tau concentration was measured in plasma samples using Quanterix total tau SIMOA Kit (101552) before and after IP (Extended Data Fig. 8). Results from MS showed high significant correlations with SIMOA measurements of p-tau181, p-tau217 and p-tau231 (only available epitopes) in TRIAD samples (Extended Data Fig. 9). The method was validated with a discovery cohort that included plasma from 24 participants (12 AD and 12 controls) in the H70 clinical studies (Institute of Neuroscience and Physiology, University of Gothenburg) (Extended Data Fig. 10). Supplementary Table 9 shows the differences in FCs when the phosphorylated peptides were normalized only with their respective standards or with the heavy standards plus the non-phosphorylated peptides.

### Brain imaging

Amyloid and tau imaging were obtained using [^18^F]AZD4694 (40–70 min after injection) and [^18^F]MK6240 (90–110 min after injection) PET, respectively^37,38^. PET scans were acquired with a Siemens High Resolution Research Tomograph (Siemens Medical Solutions), and imaging data were processed, in conjunction with each individualʼs magnetic resonance imaging, as previously described^37–39^, using the cerebellar gray matter and the inferior cerebellar gray matter as reference regions for Aβ and tau PET SUVR calculation, respectively. Amyloid status was based on visual reading of [^18^F]AZD4694 PET by two neurologists blinded to clinical diagnosis, and the global amyloid SUVR refers to the average SUVR of the precuneus, cingulate, inferior parietal, medial prefrontal, lateral temporal and orbitofrontal cortices. For tau PET, a global index of tau pathology was given by the average SUVR in the meta-ROI region. For the in vivo classification of Braak stages, average tau PET SUVR was estimated for the transentorhinal (Braak I–II), limbic (Braak III–IV) and isocortical (Braak V–VI) cortices, allowing for the determination of a regional positivity status, which was then applied in an ordinal logistic regression to determine one’s Braak stage classification, as previously described^39^.

### Statistics and reproducibiliy

The R statistical software package (4.0.0) was used to perform non-imaging statistical analyses. Chi-square or Fisher tests were conducted for categorical variables, and *t*-tests or analysis of variance (ANOVA) were conducted for continuous variables when appropriate. Correlations between biomarkers were assessed with Spearman rank tests. As mentioned above, participants were grouped according to cognitive and amyloid statuses. In addition, amyloid PET global SUVRs were split into quartiles, which was also used to segregate participants into groups (Q1 = (−Inf, 1.29); Q2 = (1.29, 1.7); Q3 = (1.7, 2.45); Q4 = (2.45, Inf)). Biomarker *z*-scores and mean FCs were calculated using average values of the CU− as reference. Linear models had the plasma biomarkers as dependent variables and tested the effect of imaging biomarkers, as independent factors, both in isolation and in an additive manner, and had age and sex as covariates. Adjusted R^2^ values and adjusted AIC were used to compare the fit of these regression models. Biomarker values that were *z*-scored were plotted against amyloid PET (centiloids) and Braak stage classification, and curves were smoothed using the LOESS method. Finally, voxel-wise regression analyses were conducted on Rminc to evaluate the associations between the different plasma markers and in vivo Aβ PET and tau PET, adjusted for age and sex. Random field theory^40^ was applied on the *t*-parametric maps to correct for multiple comparisons. No a priori sample size calculation was performed. In addition, not all biomarkers were normally distributed, but, although no data transformation was applied, normality of the ANOVA/linear model residuals was visually inspected using quantile plots.

### Reporting Summary

Further information on research design is available in the Nature Portfolio Reporting Summary linked to this article.

## Supplementary information

Supplementary Tables 1–9

Reporting Summary

## Figures and Tables

**Extended Data Fig. 5:**
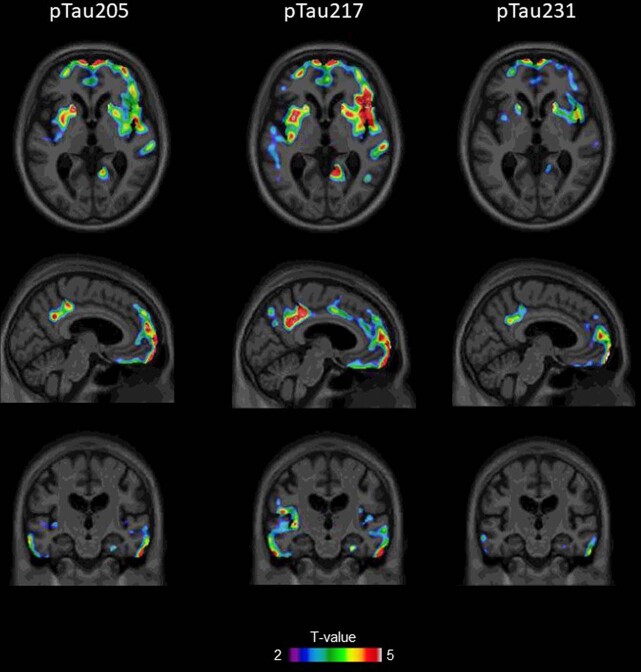
Voxel-wise associations of p-tau205, p-tau217 and p-tau231 with Amyloid PET uptake. Adjusted for multiple comparisons maps depict the results of the association between plasma biomarkers and Amyloid PET at the voxel level (n=51). Voxels with T-value>3 have a significant association between the variables evaluated. P-tau181 is not included in the figure since it did not survive adjustment for multiple comparisons.

**Extended Data Fig. 7:**
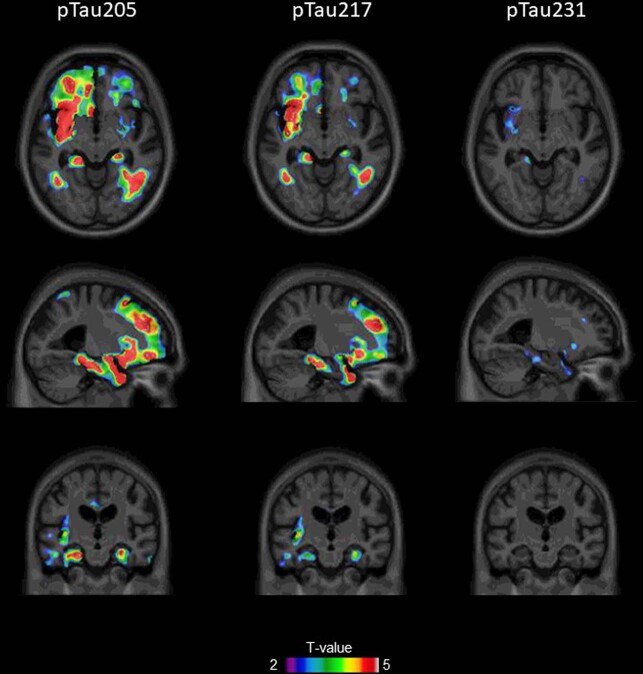
Voxel-wise associations of p-tau205, p-tau217 and p-tau231 with Tau PET uptake. Adjusted for multiple comparisons maps depict the results of the association between plasma biomarkers and Amyloid PET at the voxel level (n=51). Voxels with T-value>3 have a significant association between the variables evaluated. P-tau181 is not included in the figure since it did not survive adjustment for multiple comparisons.

## Data Availability

This study includes no data deposited in external repositories. Anonymized data can be shared upon reasonable request from a qualified academic investigator for the sole purpose of replicating procedures and results presented in the article, as long as data transfer agrees with local legislation and with the local ethical review board of each cohort, which must be regulated in a material/data transfer agreement.

## Code availability

This study does not use customized algorithms or software.
