# Supplementary material for: Mass spectrometric simultaneous quantification of tau species in plasma shows differential associations with amyloid and tau pathologies
Source: Nat Aging. 2023 Apr 27;3(6):661–9. doi: 10.1038/s43587-023-00405-1 (PMC10275761; doi:10.1038/s43587-023-00405-1)
Supplement: Supplementary file 1 — Supplementary Tables 1–9 [file 43587_2023_405_MOESM1_ESM.pdf]

nature aging

Letter

https://doi.org/10.1038/s43587-023-00405-1

Mass spectrometric simultaneous quantification of tau species in plasma shows differential associations with amyloid and tau pathologies

In the format provided by the authors and unedited

TABLE OF CONTENTS

Supplementary Table 1. Plasma levels and demographics of participants in Cohort 1.................. 2

Supplementary Table 2. Demographics, plasma and imaging biomarker levels of participants in Cohort 2... 3

Supplementary Table 3. Plasma biomarker levels in non-AD participants in Cohort 2. 4

Supplementary Table 4. Correlations between biomarkers.. 4

Supplementary Table 5. Biomarker performance. 5

Supplementary Table 6. Tryptic tau endogenous peptides targeted in the study. 5

Supplementary Table 7. Heavy standards used for the nomalization of quantified peptides......... 6

Supplementary Table 8. Coefficient of variation (CV) of each targeted peptide........................... 6

Supplementary Table 9. Fold-changes in the levels of each phosphorylated peptide in the Alzheimer´s disease group compared to controls (Discovery cohort, H70 studies) alone or the ratio of the phosphorylated peptide vs non-phosphorylated... 6

Supplementary Table 1. Plasma levels and demographics of participants in Cohort 1. Biomarker values and age are given in median fmol/mL (IQR) and mean (SD). Units: plasma biomarkers (fmol/ml), CSF biomarkers (pg/ml) and age (years). Abbreviations: AD (Alzheimer´s disease), MCI (Mild-Cognitively Impaired), F (female), IQR (Interquartile Range), SD (Standard Deviation).

		**Control (N=23)**	**AD MCI (N=24)**	**AD (N=27)**	**MCI other (N=50)**	**Other dementia (N=33)**
**P-tau181**	Median (IQR)	0.0272 (0.0131)	0.0407 (0.0146)	0.0426 (0.0115)	0.0299 (0.0161)	0.0304 (0.0126)
	Mean (SD)	0.0274 (0.00977)	0.0458 (0.0189)	0.0454 (0.0171)	0.0316 (0.0121)	0.0333 (0.0154)
**Tau 212-221**	Median (IQR)	0.221(0.0549)	0.288(0.119)	0.283(0.137)	0.252(0.146)	0.250(0.112)
	Mean (SD)	0.224(0.0922)	0.314(0.0969)	0.298(0.118)	0.270(0.122)	0.259(0.105)
**P-tau217**	Median (IQR)	0.00213 (0.00213)	0.00553 (0.00418)	0.00870 (0.00775)	0.00245 (0.00239)	0.00260 (0.00255)
	Mean (SD)	0.00388 (0.00494)	0.00694 (0.00602)	0.0104 (0.00674)	0.00350 (0.00292)	0.00442 (0.00558)
**P-tau199**	Median (IQR)	0.00285 (0.00543)	0.00255 (0.00475)	0.00390 (0.00475)	0.00110 (0.00360)	0.00140 (0.00380)
	Mean (SD)	0.00372 (0.00446)	0.00687 (0.0159)	0.00382 (0.00350)	0.00208 (0.00242)	0.00222 (0.00275)
**P-tau202**	Median (IQR)	0.0397 (0.0385)	0.0702 (0.0788)	0.0469 (0.0420)	0.0334 (0.0337)	0.0451 (0.0211)
	Mean (SD)	0.0488 (0.0416)	0.0827 (0.0633)	0.0623 (0.0438)	0.0472 (0.0439)	0.0402 (0.0205)
**P-tau205**	Median (IQR)	0 (0.000115)	0.000590 (0.00216)	0.00137 (0.00191)	0(0.000843)	0.000300 (0.000910)
	Mean (SD)	0.00167 (0.00545)	0.00113 (0.00132)	0.00201 (0.00220)	0.00193 (0.00760)	0.00102 (0.00260)
**P-tau231**	Median (IQR)	0.00340 (0.00363)	0.00885 (0.0127)	0.0166 (0.0155)	0.00470 (0.00770)	0.00410 (0.00780)
	Mean (SD)	0.00477 (0.00476)	0.0129 (0.0121)	0.0164 (0.0104)	0.00593 (0.00581)	0.00756 (0.0101)
**CSF Aβ42/40**	Median (IQR)	6.49 (2.33)	16.11 (5.57)	15.39 (5.54)	9.58 (3.80)	8.46 (3.76)
	Mean (SD)	7.46 (2.41)	17.31 (5.19)	16.96 (3.91)	9.54 (2.63)	8.93 (2.73)
**CSF p-tau**	Median (IQR)	13.30 (4.00)	24.90 (19.48)	26.77 (17.72)	14.10 (9.70)	17.80 (10.80)
	Mean (SD)	13.45 (2.65)	29.62 (22.97)	40.50 (37.40)	16.53 (6.95)	21.39 (15.01)
**CSF t-tau**	Median (IQR)	159.00 (46.24)	241.18 (173.86)	321.00 (208.00)	165.50 (94.33)	217.00 (127.00)
	Mean (SD)	165.81 (29.01)	301.69 (200.19)	661.86 (1503.33)	192.94 (71.97)	263.40 (170.75)
**MMSE**	Median (IQR)	27.00 (4.00)	27.00 (2.25)	19.00 (5.50)	25.00 (3.75)	23.00 (7.00)
	Mean (SD)	27.17 (2.18)	27.08 (2.20)	18.63 (4.42)	24.36 (4.10)	21.27 (5.18)
**Age**	Median (IQR)	63.00 (12.00)	72.50 (10.25)	68.00 (13.50)	66.00 (10.75)	70.00 (9.00)
	Mean (SD)	62.26 (8.52)	72.25 (8.28)	68.93 (9.19)	66.14 (8.34)	69.61 (7.24)
**Sex (F)**		13 (56.5%)	13 (54.2%)	19 (70.4%)	34 (68.0%)	17 (51.5%)

Supplementary Table 2. Demographics, plasma and imaging biomarker levels of participants in Cohort 2.
Biomarker values and age are given in median (IQR) and mean (SD). Units: plasma biomarkers (fmol/ml), imaging biomarkers (SUVR) and age (years).Abbreviations: CU- (Cognitively Unimpaired amyloid PET negative), CU+ (Cognitively Unimpaired amyloid PET positive), CI+ (Cognitively Impaired amyloid PET positive) and CI- (Cognitively Impaired amyloid PET negative), F (female), SUVR (Standard uptake value ratio), IQR (Interquartile Range), SD (Standard Deviation).

		**CU-<br>(N=18)**	**CU+<br>(N=8)**	**CI+<br>(N=18)**	**CI-<br>(N=7)**
**P-tau181**	Median (IQR)	0.0431(0.0172)	0.0585(0.0290)	0.0585(0.0440)	0.0611(0.0314)
	Mean (SD)	0.0469(0.0170)	0.0580(0.0205)	0.0661(0.0291)	0.0671(0.0425)
**Tau 212-221**	Median (IQR)	0.467(0.142)	0.583(0.280)	0.451(0.256)	0.541(0.0843)
	Mean (SD)	0.495(0.141)	0.551(0.200)	0.488(0.229)	0.527(0.125)
**P-tau217**	Median (IQR)	0.00285(0.00268)	0.00758(0.00609)	0.0141(0.0181)	0.00600(0.00423)
	Mean (SD)	0.00361(0.00223)	0.00739(0.00424)	0.0167(0.0109)	0.00506(0.00294)
**Tau 195-209**	Median (IQR)	0.625(0.176)	0.778(0.379)	0.714(0.384)	0.631(0.108)
	Mean (SD)	0.635(0.141)	0.724(0.267)	0.717(0.347)	0.644(0.139)
**P-tau199**	Median (IQR)	0.00595(0.00340)	0.00650(0.00560)	0.00600(0.00458)	0.00860(0.00335)
	Mean (SD)	0.00579(0.00275)	0.00813(0.00435)	0.00692(0.00425)	0.00856(0.00309)
**P-tau202**	Median (IQR)	0.0125(0.00339)	0.0139(0.00943)	0.0126(0.00600)	0.0145(0.00458)
	Mean (SD)	0.0127(0.00431)	0.0157(0.00653)	0.0130(0.00666)	0.0156(0.00415)
**P-tau205**	Median (IQR)	0.000315(0.000430)	0.000890(0.000698)	0.00206(0.00278)	0.000870(0.000585)
	Mean (SD)	0.000451(0.000356)	0.000904(0.000650)	0.00199(0.00160)	0.000867(0.000585)
**P-tau231**	Median (IQR)	0.00785(0.00915)	0.0215(0.0135)	0.0247(0.0288)	0.0150(0.0118)
	Mean (SD)	0.0105(0.00804)	0.0187(0.00928)	0.0288(0.0182)	0.0192(0.0172)
**Tau PET**	Median (IQR)	0.817(0.140)	0.845(0.183)	1.97(1.48)	0.826(0.144)
	Mean (SD)	0.828(0.0887)	0.885(0.131)	1.92(0.792)	0.840(0.124)
	Missing	1 (5.6%)	0 (0%)	0 (0%)	0 (0%)
**Amyloid PET**	Median (IQR)	1.20(0.183)	1.88(0.205)	2.53(0.318)	1.37(0.165)
	Mean (SD)	1.26(0.122)	1.95(0.326)	2.53(0.443)	1.40(0.177)
**Sex**	female	9 (50.0%)	4 (50.0%)	9 (50.0%)	4 (57.1%)
	male	9 (50.0%)	4 (50.0%)	9 (50.0%)	3 (42.9%)
**Age**	Median (IQR)	66.3(47.4)	72.2(4.03)	72.2(9.56)	76.5(3.54)
	Mean (SD)	54.6(24.1)	72.3(2.86)	71.5(5.99)	75.7(5.24)

Supplementary Table 3. Plasma biomarker levels in non-AD participants in Cohort 2. Biomarker values are given in median (IQR) and mean (SD) (fmol/mL). Abbreviations: FTD, Fronto-temporal Dementia), PSP, (Progressive Supranuclear Palsy), IQR (Interquartile Range), SD (Standard Deviation).

		FTD (N=4)	PSP (N=2)
**P-tau181**	Median (IQR)	0.0345(0.0225)	0.0853(0.0270)
	Mean (SD)	0.0534(0.0392)	0.0853(0.0382)
**Tau 212-221**	Median (IQR)	0.476(0.109)	0.603(0.139)
	Mean (SD)	0.466(0.0785)	0.603(0.196)
**P-tau217**	Median (IQR)	0.00228(0.00184)	0.00370(0.000600)
	Mean (SD)	0.00259(0.00141)	0.00370(0.000849)
**Tau 195-209**	Median (IQR)	0.556(0.162)	0.737(0.123)
	Mean (SD)	0.553(0.110)	0.737(0.174)
**P-tau199**	Median (IQR)	0.00555(0.000475)	0.00930(0.00210)
	Mean (SD)	0.00593(0.000785)	0.00930(0.00297)
**P-tau202**	Median (IQR)	0.0131(0.00246)	0.0177(0.00338)
	Mean (SD)	0.0145(0.00436)	0.0177(0.00477)
**P-tau205**	Median (IQR)	0.000245(0.0000500)	0.000850(0.000430)
	Mean (SD)	0.000220(0.0000753)	0.000850(0.000608)
**P-tau231**	Median (IQR)	0.00705(0.00455)	0.0201(0.00805)
	Mean (SD)	0.00950(0.00609)	0.0201(0.0114)

Supplementary Table 4. Correlations between biomarkers.
Cross-correlation matrix showing Spearman rho correlation values between plasmatic and imaging biomarkers. Significant correlations are indicated: * $p<0.05$, ** $p<0.01$, ***$p<0.001$ (two-sided).

	**pTau217**	**pTau199**	**pTau202**	**pTau181**	**pTau205**	**pTau231**	**Tau212-221**	**Tau195-209**	**Tau PET**	**Amyloid PET**
**pTau217**	1									
**pTau199**	0.46***	1								
**pTau202**	0.49***	0.87***	1							
**pTau181**	0.72***	0.68***	0.81***	1						
**pTau205**	0.82***	0.66***	0.70***	0.80***	1					
**pTau231**	0.86***	0.68***	0.73***	0.90***	0.89***	1				
**Tau212-221**	0.55***	0.78***	0.86***	0.75***	0.74***	0.70***	1			
**Tau195-209**	0.70***	0.69***	0.81***	0.80***	0.81***	0.78***	0.95***	1		
**Tau PET**	0.58***	0.10	0.07	0.24	0.49***	0.43**	0.09	0.23	1	
**Amyloid PET**	0.70***	0.13	0.17	0.42**	0.52***	0.60***	0.13	0.29*	0.68***	1

Supplementary Table 5. Biomarker performance.

The area under the Receiver Operating characteristic curves (AUC-ROC) and the 95% confidence intervals are reported below to indicate the ability of plasma p-tau181, p-tau205, p-tau217 and p-tau231 to distinguish participants grouped according to their amyloid PET status or Braak stages.

	**Aβ PET positive vs negative**	**Braak I-IV vs Braak V-VI**
**pTau181**	0.65 95% CI: 0.50-0.81	0.64 95% CI: 0.40-0.87
**pTau205**	0.72 95% CI: 0.57-0.86	0.81 95% CI: 0.58-1
**pTau205/Tau195-209**	0.75 95% CI: 0.61-0.89	0.84 95% CI: 0.63-1
**pTau217**	0.85 95% CI: 0.74–0.95	0.81 95% CI: 0.59-1
**pTau217/Tau212-221**	0.94 95% CI: 0.87-1	0.96 95% CI: 0.9-1
**pTau231**	0.76 95% CI: 0.63–0.90	0.72 95% CI: 0.48-0.96

Supplementary Table 6. Tryptic tau endogenous peptides targeted in the study.

Respective dominant charge state, monoisotopic m/z value, optimal normalized collision energy for higher energy collision-induced dissociation (NCE) and optimal compensation voltage (CV) used for FAIMS for each peptide are shown.

**Phospho site**	**Peptide aa positions**	**Target peptide sequence**	**Charge state**	***m/z***	**CE [%]**	**FAIMS CV (V)**
T181	175-190	TPPAPK[pT]PPSSGEPPK	3	556.6062	25	-70
-	195-209	SGYSSPGSPGTPGSR	2	697.321	25	-50
S199	195-209	SGYS[pS]PGSPGTPGSR	2	737.3039	25	-50
S202	195-209	SGYSSPG[pS]PGTPGSR	2	737.3039	25	-50
T205	195-209	SGYSSPGSPG[pT]PGSR	2	737.3039	25	-50
-	212-221	TPSLPTPPTR	2	533.798	25	-70
T217	212-221	TPSLP[pT]PPTR	2	573.781	27	-60
T231	225-240	KVAVVR[pT]PPKSPSSAK	3	577.9887	30	-60

Supplementary Table 7. Heavy standards used for the nomalization of quantified peptides.

Respective monoisotopic m/z value, heavy labelling and spiked in concentrations are shown for each peptide. The heavy labeled aminoacids are marked in bold. Dominant charge state, optimal normalized collision energy for higher energy collision-induced dissociation (CE) and optimal compensation voltage used for FAIMS are the same as for the corresponding light peptides shown in Supplementary Table 6.

Phospho site	Peptide aa positions	Target peptide sequence	*m/z*	Spiked in per sample (fmol)
T181	175-190	TPPAPK[pT]PPSSGEPP**K**	558.6129	1
-	195-209	SGYSSPGSPGTPGS**R**	702.325	0.5
S199	195-209	SG**Y**S[pS]PGSPGTPGSR	742.3175	1
S202	195-209	SGYSSPG[pS]PGTPGS**R**	742.3081	0.5
T205	195-209	SG**Y**SSPGSPG[pT]PGSR	742.3175	0.1
-	212-221	TPSLPTPPT**R**	538.802	1
T217	212-221	TPSLP[pT]PPT**R**	578.786	0.5
T231	225-240	**K**VAVVR[pT]PPKSPSSA**K**	582.0022	1

Supplementary Table 8. Coefficient of variation (CV) of each targeted peptide.

Ratio to standard values for each Quality Control (QC) sample (n=8). QCs samples were generated by pooling plasma from healthy individuals and aliquoting in 1ml-volume samples. CVs were calculated as the standard deviation divided by the mean and expressed in percentage (%).

	p181	195-209	p199	p202	p205	212-221	p217	p231
**QC1**	0.1470	4.6961	0.0409	0.1229	0.0352	2.0081	0.0293	0.0868
**QC2**	0.1331	4.0255	0.0324	0.1019	0.0373	1.7325	0.0228	0.0798
**QC3**	0.1272	4.1781	0.0266	0.0986	0.0355	1.7186	0.0244	0.0737
**QC4**	0.1455	4.3311	0.0363	0.1183	0.0408	1.8975	0.0258	0.0784
**QC5**	0.1482	4.6931	0.0315	0.0988	0.0451	1.9108	0.0261	0.0800
**QC6**	0.1411	4.4279	0.0358	0.1052	0.0425	1.9529	0.0242	0.0935
**QC7**	0.1409	4.8416	0.0290	0.1074	0.0413	2.3541	0.0304	0.0792
**QC8**	0.1361	4.7877	0.0285	0.0956	0.0469	1.9765	0.0243	0.0677
**CV (%)**	**5.23%**	**6.71%**	**14.65%**	**9.23%**	**10.63%**	**10.14%**	**10.23%**	**9.73%**

Supplementary Table 9. Fold-changes in the levels of each phosphorylated peptide in the Alzheimer´s disease group compared to controls (Discovery cohort, H70 studies) alone or the ratio of the phosphorylated peptide vs non-phosphorylated.

	phospho peptide	phospho/195-209	phospho/212-221
**p-tau181**	1.61	1.19	0.78
**p-tau199**	1.17	0.88	0.78
**p-tau202**	1.02	0.78	0.69
**p-tau205**	4.05	3.37	2.72
**p-tau217**	1.97	1.45	1.36
**p-tau231**	2.76	2.07	2.07
