# Supplementary material for: Mass spectrometric simultaneous quantification of tau species in plasma shows differential associations with amyloid and tau pathologies
Source: Nat Aging. 2023 Apr 27;3(6):661–9. doi: 10.1038/s43587-023-00405-1 (PMC10275761; doi:10.1038/s43587-023-00405-1)
Supplement: Supplementary file 2 — Reporting Summary [file 43587_2023_405_MOESM2_ESM.pdf]

nature portfolio

Corresponding author(s): Laia Montoliu-Gaya and Kaj Blennow

Last updated by author(s): Feb 1, 2023

Reporting Summary

Nature Portfolio wishes to improve the reproducibility of the work that we publish. This form provides structure for consistency and transparency in reporting. For further information on Nature Portfolio policies, see our Editorial Policies and the Editorial Policy Checklist.

Statistics

For all statistical analyses, confirm that the following items are present in the figure legend, table legend, main text, or Methods section.

n/a	Confirmed	
☐	☒	The exact sample size (*n*) for each experimental group/condition, given as a discrete number and unit of measurement
☐	☒	A statement on whether measurements were taken from distinct samples or whether the same sample was measured repeatedly
☐	☒	The statistical test(s) used AND whether they are one- or two-sided<br>*Only common tests should be described solely by name; describe more complex techniques in the Methods section.*
☐	☒	A description of all covariates tested
☐	☒	A description of any assumptions or corrections, such as tests of normality and adjustment for multiple comparisons
☐	☒	A full description of the statistical parameters including central tendency (e.g. means) or other basic estimates (e.g. regression coefficient) AND variation (e.g. standard deviation) or associated estimates of uncertainty (e.g. confidence intervals)
☐	☒	For null hypothesis testing, the test statistic (e.g. *F*, *t*, *r*) with confidence intervals, effect sizes, degrees of freedom and *P* value noted<br>*Give P values as exact values whenever suitable.*
☒	☐	For Bayesian analysis, information on the choice of priors and Markov chain Monte Carlo settings
☒	☐	For hierarchical and complex designs, identification of the appropriate level for tests and full reporting of outcomes
☐	☒	Estimates of effect sizes (e.g. Cohen's *d*, Pearson's *r*), indicating how they were calculated

Our web collection on statistics for biologists contains articles on many of the points above.

Software and code

Policy information about availability of computer code

Data collection	Thermo Scientific Xcalibur 4.5, Thermo Scientific Tune 3.5
Data analysis	Skyline 22.2, R (version 3.6.3) and Rminc (Version 1.5.2.3) software and their publicly available packages.

For manuscripts utilizing custom algorithms or software that are central to the research but not yet described in published literature, software must be made available to editors and reviewers. We strongly encourage code deposition in a community repository (e.g. GitHub). See the Nature Portfolio guidelines for submitting code & software for further information.

Data

Policy information about availability of data

All manuscripts must include a data availability statement. This statement should provide the following information, where applicable:
- Accession codes, unique identifiers, or web links for publicly available datasets
- A description of any restrictions on data availability
- For clinical datasets or third party data, please ensure that the statement adheres to our policy

This study includes no data deposited in external repositories. Anonymized data can be shared upon reasonable request from a qualified academic investigator, for the sole purpose of replicating procedures and results presented in the article, as long as data transfer agrees with local legislation and with the local Ethical Review Board of each cohort, which must be regulated in a material/data transfer agreement.

Human research participants

Policy information about studies involving human research participants and Sex and Gender in Research.

Reporting on sex and gender	In TRIAD, sex was balanced between groups and was it accounted for in the relevant statistical analyses. In Paris Lariboisière cohort, samples were included systematically based on availability and sex was not considered a priori.
Population characteristics	Age and sex were used as covariate in the linear models performed on the data and on the imaging analysis and they were not significant in those models. The description of age and sex per groups (in each cohort) is reported in Suppl. Tables 1 and 2.
Recruitment	Participants were recruited from memory clinics and general population in the region of Montreal-Canada and Paris-France
Ethics oversight	The TRIAD study was approved by The Research Ethics Board of the Montreal Neurological Institute as well as the Faculty of Medicine Research Ethics Office, McGill University. The Paris Lariboisière cohort was approved by the ethic committee of Bichat University, Paris, France (CEERB GHU Nord n°10-037).

Note that full information on the approval of the study protocol must also be provided in the manuscript.

Field-specific reporting

Please select the one below that is the best fit for your research. If you are not sure, read the appropriate sections before making your selection.

☒ Life sciences ☐ Behavioural & social sciences ☐ Ecological, evolutionary & environmental sciences

For a reference copy of the document with all sections, see nature.com/documents/nr-reporting-summary-flat.pdf

Life sciences study design

All studies must disclose on these points even when the disclosure is negative.

Sample size	For TRIAD, samples were chosen based on the availability of fluid volume for analysis, age, clinical diagnosis, and availability of imaging data (N=51). For Paris Lariboisière, samples were systematically included based on the availability of fluid volumes for analysis (N=157). We have not calculated sample size à priori. However, based on the data from Paris cohort which is the larger cohort we have started the study with, we saw that an eta2 of 0.2 in an ANOVA testing the effect of diagnostic groups on MS p-tau181 (with MS p-tau217 the eta2=0.22). If we use this parameter to calculate the sample size needed to find the same effect on TRIAD (considering an ANCOVA model testing a group effect with 4 levels, 2 covariates, an alpha=0.05 and a power of 0.8) we would need 48 participants (or 61 with a power of 0.9). Repeating this analysis using MS p-tau217 would require us to have 43 or 55 participants, with a power of 0.8 and 0.9 respectively. Thus, despite the know limitation of sample size in the TRIAD cohort, we trust that this is enough to capture to main and most important conclusions of this study.
Data exclusions	No data were excluded from the analyses.
Replication	Replication was done by repeating the reported analysis in 2 different cohorts. All attempts at replication were successful.
Randomization	For Paris cohort, biomarker levels were compared according to the participant's diagnostic group, which was defined by clinical evaluation prior to data analysis. For TRIAD, given the smaller sample size, participants were grouped according to their cognition (unimpaired/impaired) and amyloid status (positive/negative).
Blinding	Fluid analysis was done blind to any information regarding to clinical or imaging status.

Reporting for specific materials, systems and methods

We require information from authors about some types of materials, experimental systems and methods used in many studies. Here, indicate whether each material, system or method listed is relevant to your study. If you are not sure if a list item applies to your research, read the appropriate section before selecting a response.

Materials & experimental systems

n/a	Involved in the study
☐	☒ Antibodies
☒	☐ Eukaryotic cell lines
☒	☐ Palaeontology and archaeology
☒	☐ Animals and other organisms
☒	☐ Clinical data
☒	☐ Dual use research of concern

Methods

n/a	Involved in the study
☒	☐ ChIP-seq
☒	☐ Flow cytometry
☒	☐ MRI-based neuroimaging

Antibodies

Antibodies used	Tau12 (Purified anti-Tau, 6-18 Antibody, 806501, Biolegend)/ HT7 (Tau Monoclonal Antibody, MN1000, Thermofisher)/ BT2 (Tau Monoclonal Antibody, MN1010, Thermofisher
Validation	Tau12 (https://www.biolegend.com/en-us/search-results/purified-anti-tau-6-18-antibody-11569), HT7 (https://www.thermofisher.com/antibody/product/Tau-Antibody-clone-HT7-Monoclonal/MN1000), BT2 (https://www.thermofisher.com/antibody/product/Tau-Antibody-clone-BT2-Monoclonal/MN1010)
